# Aberrant placental structure is corrected with repeated nanoparticle-mediated IGF1 treatments in a Guinea pig model of fetal growth restriction

**DOI:** 10.1016/j.tria.2025.100424

**Published:** 2025-07-25

**Authors:** Baylea N. Davenport, Rebecca L. Wilson, Alyssa A. Williams, Jaimi A. Gray, Edward L. Stanley, Helen N. Jones

**Affiliations:** a Center for Research in Perinatal Outcomes, College of Medicine, University of Florida, Gainesville, FL, USA; b Department of Physiology and Aging, College of Medicine, University of Florida, Gainesville, FL, USA; c Florida Museum of Natural History, University of Florida, Gainesville, FL, USA; d The University of Texas at Austin, Department of Earth and Planetary Sciences, Austin, TX, USA

**Keywords:** Fetal growth restriction, Placental structure, Vascular remodeling, Gene therapy, Nanoparticle, Nano-CT

## Abstract

**Background:**

Fetal growth restriction (FGR) is most commonly due to placental insufficiency. There are currently no treatments for placental insufficiency or FGR, and the only intervention is iatrogenic pre-term delivery. We have previously shown efficacy of repeated placental nanoparticle-mediated *insulin-like 1 growth factor* (*IGF1*) treatment in improving placental efficiency (increased fetal-placental weight ratio) and correcting fetal growth in a maternal nutrient restriction (MNR) guinea pig model of FGR. We hypothesize placenta structural changes (reduced exchange area, altered vascular structure) that we and others have previously shown in the FGR/MNR placenta which lead to deficits in placental function are mitigated by our repeated nanoparticle-mediated *hIGF1* treatment.

**Methods:**

Here we investigate the structural remodeling of the placenta in a maternal nutrient restriction (MNR) guinea pig model following 3 repeated intraplacental injections of nanoparticle-mediated *hIGF1* that may underpin the published improvements in placental efficiency and fetal growth. Using immunohistochemistry and Dice-CT we investigated the micro- and macrovasculature changes of the placenta structure to identify changes in FGR and treatment.

**Results:**

Sham-treated MNR placentas displayed disorganized microvasculature labyrinthine exchange areas with a reduction in placental capillary number and an increase in the volume of the placenta macrovasculature. Repeated nanoparticle-mediated *hIGF1* treatment, however, resulted in an improved exchange area with normalized placental capillary number and macrovasculature volume.

**Conclusions:**

This data demonstrates repeated nanoparticle-mediated *hIGF1* delivery corrects aberrant placenta structure during FGR and placental insufficiency likely leading to improved gas exchange and transfer of nutrients to the fetus restoring fetal growth.

## 1. Introduction

Fetal growth restriction (FGR) affects 10 % of humans worldwide and is defined as a fetus failing to reach their growth potential [1–3]. While diagnosis criteria varies between regions, the most commonly accepted criteria according to ACOG (American College of Obstetricians and Gynecologists) guidelines is an estimated fetal weight (EFW) or abdominal circumference below the 10th percentile for gestational age. FGR can lead to stillbirth and iatrogenic preterm delivery, with FGR neonates having increased risk of developing numerous comorbidities linked to aberrant fetal programming [4]. FGR is associated with life-long consequences stemming from the developmental origins of health and disease (DOHaD) including cognitive delays/deficits, cardiovascular disorders, and metabolic disorders [5,6]. Most often, the cause of this condition is due to placental insufficiency: the placenta’s failure to supply sufficient oxygen and nutrients to the fetus for proper growth and development [7,8].

Placental functions include gas/oxygen exchange, immune barrier, endocrine function, nutrient transfer, and waste excretion and is vital for fetal growth and development. Proper blood flow into, and structure of, the placenta is critical to deliver all necessary oxygen and nutrients from the maternal circulation, across the syncytium and villous core to the fetal circulation. The macrovasculature structures, or the stem-like structures branching from either the uteroplacental vessels (of the maternal circulation) and the umbilical cord (the fetal circulation) lead into highly branched microvascular exchange areas to support the increasing transfer and exchange throughout pregnancy. Insufficient placentas of growth restricted fetuses display abnormalities in both the utero-placental circulation and the feto-placental circulation, impeding these pivotal processes [9,10]. Human FGR studies show maternal vascular abnormalities in spiral artery remodeling within the uterus leading to increased resistance shown by uterine artery doppler [9–13]. The fetoplacental vascular network also displays increased resistance with underdeveloped terminal villi/hypovascularity [14–16]. These aberrant vasculatures restrict blood flow and reduce gas exchange, waste exchange, and nutrient transport in the microvasculature exchange area [9,10,14].

Guinea pigs offer one of the most useful models to study placental development and fetal growth restriction due to their developmental similarities to humans [17]. Like humans, guinea pigs have invasive, haemomonochorial, discoid placentas. Similar to the human placenta which is organized into cotyledons each being supplied by a single spiral artery from the maternal circulation, the guinea pig placenta has distinct lobules of circulatory exchange with microvasculature bringing maternal blood to the syncytial lined maternal lacunae of each lobules exchange region. [12]. Similar to the chorionic plate vessels and stem villi vessels in the human, the feto-placental circulation macrovasculature of each lobule branches to form a network of microvasculature. Bloodflow within the maternal lacunae and apposing macrovasculature carrying fetal blood is counter-current to maximize the area of nutrients and oxygen exchange [18]. Guinea pig fetuses recapitulate human developmental milestones throughout gestation and after birth making them an excellent candidate to study both placental and fetal development [17,19]. To model FGR and placental insufficiency, the well-established maternal nutrient restriction (MNR) model is employed [20]. The MNR model uses a reduction in food intake (70 % of Control) for the mother to increased stress/cortisol [19–21]. Roberts et al. previously showed at both mid and late gestation, the MNR guinea pig placenta had reduced exchange surface area and increased barrier thickness, recapitulating the dysregulated microvasculature exchange area seen in human placentas [22]. This model has been consistently shown to induce insufficient placental development and function, and impact similar signaling cascades that lead to reduced growth in human cases of FGR [20–22].

The present study employs the guinea pig MNR model to study the effects of a novel gene therapy for the correction of placental insufficiency and in turn FGR [23,24]. The insulin-like growth factor (IGF) axis is one of the key signaling cascades shown to be downregulated in placentas of FGR humans and animal models [25,26]. IGF1 is produced by placental trophoblast throughout gestation with numerous roles in regulating nutrient transport, proliferation, and angiogenesis [27,28]. Using ultrasound guided intraplacental injection of nanoparticles containing the *hIGF1* gene under the control of a trophoblast-specific primer, it was previously demonstrated [29–31] that increased placental capillary volume density and reduced interhaemal distance between maternal and fetal blood supply at mid-pregnancy, which may facilitate increased gas exchange and nutrient transfer [30]. With three injections starting after establishment of FGR (each 8 days apart) fetal growth was corrected to Control weight by term [23].

The overall aim of this study was to investigate the structural changes of the placenta labyrinth in a model of placental insufficiency and assess the impact of repeated nanoparticle-mediated trophoblast-specific *hIGF1* treatments. The hypothesis was that *hIGF1* treatment improved placenta efficiency by restoring placental labyrinth vasculature structure (both micro and macro) for improved blood flood, gas exchange, and access of syncytium for nutrient uptake.

DiceCT (diffusible iodine contrast enhanced computed tomography) has been a critical tool enabling comparative biologists to increase their knowledge on taxonomy and museum archivists to create digital databases of museum specimens. The oVert (OpenVertebrate) project was designed to provide open access digital 3D vertebrate anatomy models to researchers and the public using CT for skeletal models and diceCT imaging for soft tissue models [32,33]. Throughpartnership with the oVert team at the Florida Museum of Natural History this technology was used for pre-clinical work on FGR. Using this technique, the macrovasculature of the guinea pig placenta was 3D rendered to understand the vascular changes within the placental lobules during placental insufficiency and following nanoparticle-mediated *h*IGF1 treatment. From this segmentation the size (volume/surface area) of the lobule vasculature networks was evaluated to understand the potential impact on blood flow/gas exchange in MNR and immunohistochemistry was used to evaluate the labyrinth maternal lacunae and microvascular changes following treatment.

## 2. METHODS

### 2.1. Nanoparticle synthesis

Nanoparticles were complexed using a lyophilized non-viral PHPMA_115_-b-PDMEAMA_115_ co-polymer reconstituted in water combined with plasmid containing the *hIGF1* gene under the control of a trophoblast-specific promoter, *CYP19A1.* Fifty μg of plasmid was suspended in a 200 μL volume solution at room temperature. Detailed methods of copolymer synthesis and nanoparticle formation can be found in Wilson et al., 2022 [30].

### 2.2. Fetal growth restriction & placental insufficiency model

Animal care and usage was approved by the Institutional Animal Care and Usage Committee at the University of Florida (Protocol #202011236). Female Dunkin-Hartley guinea pigs (Dams) were purchased from Charles River Laboratories (Wilmington, MA) at 500–550 g (~8–9 weeks of age). Animals were housed in an environmentally controlled room (22 °C/72 °F, 50 % humidity 12 h light-dark cycle). Upon arrival, food (LabDiet diet 5025: 27 % protein, 13.5 % fat, and 60 % carbohydrate as % of energy) and water were provided ad libitum. Guinea pigs were acclimatized for 2 weeks prior to being assigned to either ad libitum diet (termed Control: n = 6) or maternal nutrient restriction (MNR) diet (n = 12). Assignment was done by ranking animals heaviest to lightest and systematically assigning them to each group for even biological weight distribution. MNR diet consists of a 70 % food intake diet based on kilogram of body weight of Control from 4 weeks pre-mating through mid-pregnancy (GD35), then increased to 90 % food intake through term to maintain pregnancy as previously published [30]. Estrous cycles were monitored starting at time of diet and guinea pigs were time mated accordingly.

### 2.3. Intraplacental nanoparticle injections, animal sacrifice, and tissue collection

Ultrasound guided intraplacental nanoparticle delivery was performed a total of 3 times, each 8 days apart from mid pregnancy till term (GD36,44,52 ± 3) with guinea pigs being sacrificed 8 days after the final injection (GD60 ± 3). One placenta per litter was injected with either nanoparticles containing Cyp19A-*hIGF1* (MNR + IGF1 n = 6) or a non-expressing plasmid sham nanoparticle (Control n = 6, MNR n = 6). In the MNR + IGF1 dams, placentas were separated based on receiving a direct injection of nanoparticle-mediated *hIGF1* “MNR + IGF1 (Direct Injection)” or by being indirectly exposed to circulating residual nanoparticle-mediated *hIGF1* “MNR + IGF1 (Indirect Exposure)” which was further confirmed by expression levels of *hIGF1* in each placenta via qPCR [23]. Dams were sacrificed via carbon dioxide asphyxiation followed by cardiac puncture and exsanguination. Fetuses (Control: n = 8 female and n = 11 male, MNR: n = 5 female and 11 male, MNR + IGF1: n = 6 female and 10 male) and the delivered placenta (placenta, sub-placenta, and decidua) were removed from the uterus and weighed. Fetal sex was noted at this time by examination of the gonads. Here it was noted that by random chance only male fetuses received direct injections (as fetal sex cannot be reliably determine at time of first injection). Blood was collected via cardiac puncture from dams and fetuses at time of sacrifice. To reduce bias, all subsequent analyses were performed blinded. For in-depth synopsis of injections, litter size, etc see Davenport, Wilson et al., 2024 [23].

### 2.4. Placenta preparation

At time of sacrifice all placentas were halved (each half included placenta, sub-placenta, and decidua still attached). One half was fixed in 4 % Paraformaldehyde for 48 h and washed in PBS for 24 h before being placed in increasing ethanol concentrations until reaching 70 % ethanol. Placenta specimen remained in 70 % ethanol until staining for diceCT (diffusible iodine contrast enhanced computed tomography) or being processed for histological analysis [34]. Remaining halves were used for protein and RNA analysis not included in this manuscript.

### 2.5. Iodine staining

In placentas analyzed for DiceCT, iodine staining was performed by combining 15 % Lugol’s iodine (Sigma) with 2x Sorenson’s buffer [32, 35]. Placentas were completely submerged in 1.25 % Lugol’s solutions for 7 days, followed by 2.5 % Lugol’s solution for 10 days. DiceCT introduces X-ray opaque iodine into soft tissues via diffusion, and iodine in the form of triiodide binds to lipids and carbohydrates abundant in soft tissue structures. The staining process was considered complete when the iodine in the container remained a deep red color indicating no more diffusion into the tissue. Any tissues considered overstained (iodine diffusing back out of tissue) were placed briefly into a water bath to allow the iodine to partially diffuse back out of the tissue. Number of samples from Control group: Dams: n = 2, placentas n = 6, Number of samples from MNR group: Dams: n = 3 female, placentas n = 9, Number of samples from MNR + IGF1 group: Dams: n = 3, placentas n = 9.

### 2.6. DiceCT scanning

Placentas were removed from iodine, individually wrapped in packing foam, and placed in small plastic bags. Plastic bags were rolled and placed in small plastic tubes to reduce movement throughout the scans. Space was left empty in the bottom of the plastic bag below the specimen to provide room for any residual iodine to drain for optimal scanning without scattering or saturation of the light source from pooled iodine. Aluminum wire was placed in each tube beside each specimen to serve as a material of known density for gray scale standardization at a later step in the analysis. The GE Phoenix c| tome |x m was used to perform nano-CT imaging. Scans were performed at 80 kV and 200 μA, with 1499 s exposure timing (roughly 25 min), 3x frame averaging with 1 skipped frame, a gain (sensitivity) value of S = 2, and a 0.3 mm aluminum filter with a voxel size of ~0.016 in the x, y, and z direction. Raw tomography projections were reconstructed using GE Datos R software (General Electric, version 2.8.2) to generated tiff (tagged image file format) stacks of approximately 2000 cross-sectional images. Three-D reconstructions of placentas imported into Volume Graphics (VG) Studio Max version 2024.2.1 (Volume Graphics, Heidelberg, Germany) for segmentation and analysis.

### 2.7. DiceCT analysis/segmentation

Three-D reconstructions were imported into VG Studio Max and aluminum wires were used to standardize gray scale values. The “paint and segment” machine learning tool was used to identify packaging/air surrounding the tissue, placental tissue, and the macrovasculature within the placenta. To avoid overtraining the machine learning models, training was performed for no longer than 30 min. Once segmentation was rendered from the machine learning, the “draw” function was used to manually clean-up any identification errors from knowledge of placenta morphology.

### 2.8. Immunohistochemistry

Remaining fixed placentas not used for DiceCT were processed and paraffin embedded according to standard protocol. Five μm thick sections were cut and slide-mounted for double-label immunohistochemistry (IHC). Sections were de-waxed and rehydrated using histo-clear and ethanol following standard protocols. Antigen retrieval was performed with 0.03 % protease (Sigma) at 37 °C for 15 min. Endogenous peroxidase activity was blocked with 3 % hydrogen peroxide for 30 min before blocking with serum-free protein block (Dako) for 10 min. Placental capillary endothelium was identified using anti-vimentin (Dako Vim3B4, 1:100 [0.5 μg/ml]) and placental trophoblasts were identified using anti-pan cytokeratin (Sigma C2562, 1:200 [10 μg/ml]). Anti-vimentin antibody was diluted in 10 % guinea pig serum with 1 % BSA and applied to slides overnight at 4 °C. Sections were washed in PBS before biotinylated anti-mouse IgG secondary antibody (Vector BA-9200, 1:200 [10 μg/ml]) was diluted in 10 % guinea pig serum with 1 % BSA and applied to sections for 30 min. Antibody signal was amplified using ABC (Vector) for 30 min and visualized with DAB with nickel (3,3′-diaminobenzidine tetrahydrochloride, Vector) to create a black precipitate. This process from protein block onward was repeated with anti-pan cytokeratin (2 h primary at room temperature) and visualized with DAB excluding nickel to form brown precipitate. Nuclei were counterstained using hematoxylin and coverslips mounted using DPX mounting solution (Millipore). Sections were imaged using the Zeiss Axioscan Scanning Microscope and Zen Imaging Software v.3.5 at 40× magnification. N numbers: Control: Dams: n = 2, placentas: n = 7; MNR: Dams: n = 3, placentas: n = 7; MNR + IGF1: Dams: n = 2, placentas n = 8.

### 2.9. Morphometry analysis

Morphometric analysis was performed using 10 random 40x fields of view of double labeled IHC. Trophoblast and maternal blood space volumes were calculated using point counting with an isotropic L-36 Merz grid via Fiji ImageJ v.2.14.0/1.54f [36]. Counting was performed as described in Roberts et al., 2001 manuscript [22]. Placental capillary number was calculated by counting capillary lumen per field of view, and an average was calculated for each placenta.

### 2.10. Statistical analysis

All data was analyzed using SPSS Statistics 29. Distribution assumptions were checked with a Q-Q-Plot. Statistical significance was determined using generalized estimating equations with gamma log link as the distribution/link function. Dams were considered the subject, with diet and nanoparticle-mediated *hIGF1* exposure and direct injection treated as main effects. Gestational age, sex, and litter size were set as covariates. In the sham treated Control and MNR groups, direct placental injection had no effect for any outcomes measured and was therefore removed as a main effect in these groups. Fetal sex-specific differences were not found amongst any of the data and therefore, outcomes for placentas from male and female fetuses were grouped together. Statistical significance was considered at P ≤ 0.05. Bonferroni post hoc analysis was performed for statistical significance. Results are reported as estimated marginal means ± 95 % confidence interval.

## 3. RESULTS

**Fig. 1.**
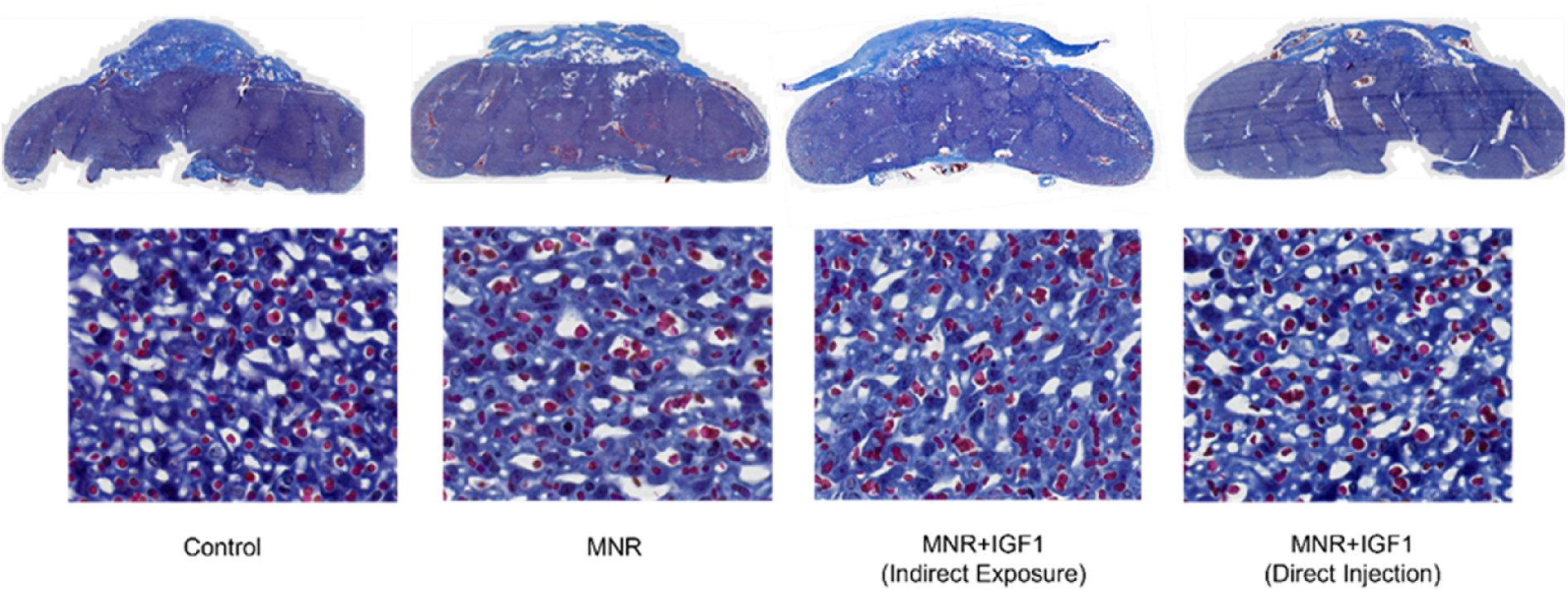
Effects of maternal nutrient restriction (MNR) and repeated *hIGF1* nanoparticle gene therapy (MNR + *hIGF1*) at end of term on gross placenta morphology. Whole scan trichrome images of placentas and sub-placenta/decidua (top), and 40x high resolution images of labyrinth area (bottom).

Maternal physiological outcomes, fetal growth outcomes, and placental weight/efficiency have been previously published [23]. Details of mating, pregnancies, and fetal weight restoration with repeated *hIGF1* nanoparticle treatment were previously described [23]. At time of sacrifice, it was determined that all placentas that received repeated direct injection of *hIGF1* nanoparticle treatment were male. As fetal sex cannot be determined at time of first injection, data from the MNR + IGF1 (Direct Injection) group only includes males, while all other groups include both sexes. No changes were seen in gross morphology of the placentas between any groups, nor hemorrhages or gross anomalies (Fig. 1).

**Fig. 2.**
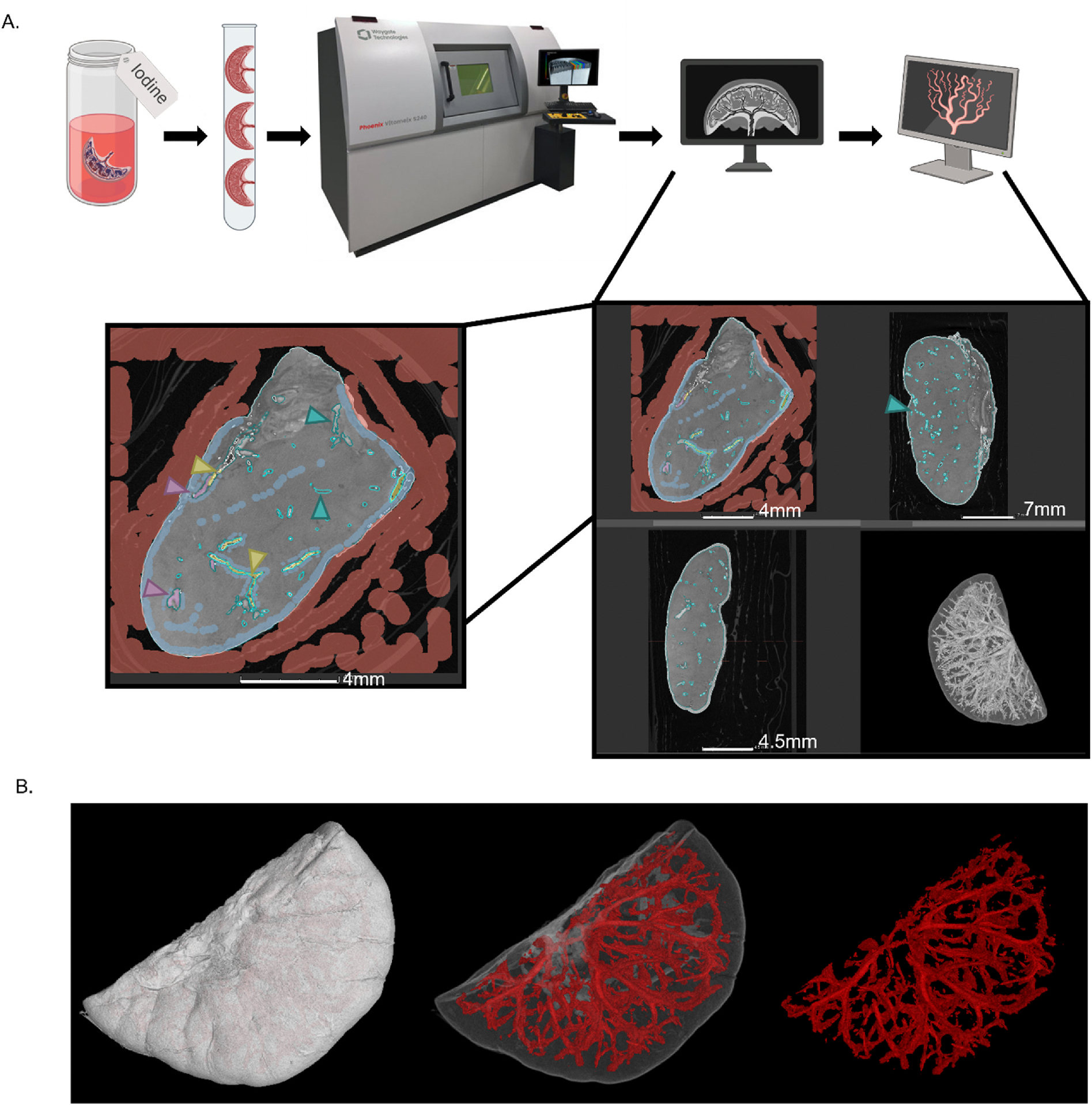
Schematic of DiceCT methodology used to create 3D rendering of placenta macrovasculature structure. **A.** Placentas were stained with iodine, packaged for stability during scanning, scanned with NanoCT to create 2D tiff stack images, and 3D rendered. VG Studio machine learning tool was used for vasculature segmentation for macrostructure modeling. Zoomed image displays machine learning training where red indicates “air/packaging” around the specimen, sky blue indicates “placenta”, purple and yellow indicate “macrovasculature”, and teal identifies areas of learned segments. **B.** Whole volume placenta 3D rendering, Macrovasculature shown within the placenta, and segmented macrovasculature.

Using DiceCT methodology and volume graphics machine learning, Control, MNR, and MNR + IGF1 placentas were 3D rendered to determine the structure of their macrovasculature (Fig. 2A). While this methodology does not differentiate between macro- and micro-structures, a resolution limit of >~13 μm was obtained when scanning and therefore could only identify the macrovasculature as seen in the rendering of the solid placenta volume and macrovasculature segmentation (Fig. 2B).

**Fig. 3.**
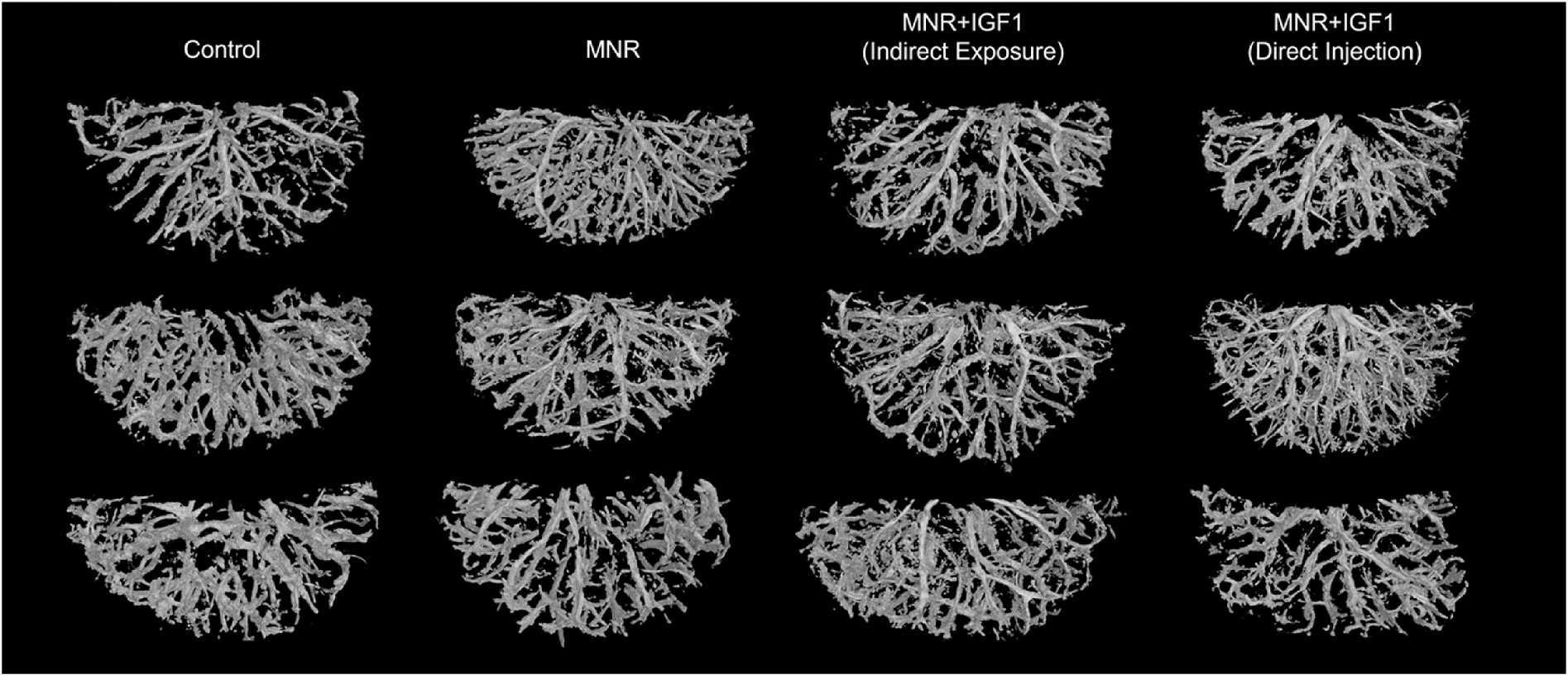
Segmentation of Control, maternal nutrient restriction (MNR), and repeated *hIGF1* nanoparticle gene therapy (MNR + *hIGF1*) placental vasculature. Three representative images from each experimental group of segmented placenta macrovasculature of male fetuses depict variety of vasculature structure between all placentas.

**Fig. 4.**
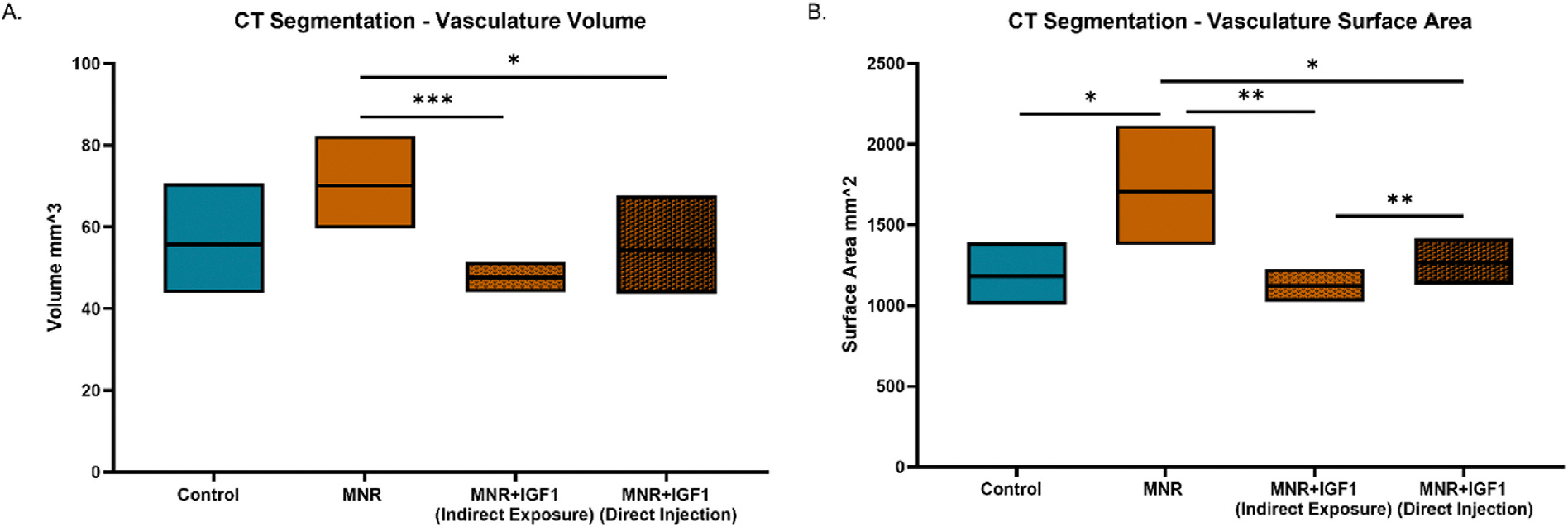
Effects of maternal nutrient restriction (MNR) and repeated *hIGF1* nanoparticle gene therapy (MNR + *hIGF1*) at end of term on placental macrostructure. **A.** Vasculature volume of placenta macrostructure decreased in MNR + IGF1 (Indirect Exposure) and MNR + IGF1 (Direct Injection), but no group was different from Control. **B.** Surface area of placenta macrostructure was increased in MNR compared to Control, MNR + IGF1 (Indirect Exposure), and MNR + IGF1 (Direct Injection). Surface area of MNR + IGF1 (Direct Injection) placentas increased from MNR + IGF1 (Indirect Exposure), but neither differed from Control. Control: Dams: n = 2, placentas n = 6, MNR: Dams: n = 3 female, placentas n = 9, MNR + IGF1: Dams: n = 3, placentas n = 9. Data are estimated marginal means ± 95 % confidence interval. *P ≤ 0.05; **P ≤ 0.01. ***P ≤ 0.001.

Vasculature 3D segmentation highlighted the complex placenta macrovasculature of maternal and fetal circulation, showing natural variation within each experimental group (Fig. 3). The size of the macrovasculature, both volume (how much space the blood can occupy) and surface area (how large the external surfaces of the vessel is), dictate how much and with what force blood flows through these structures and can provide insights into force on these vessels, back pressure. To elucidate changes in size of the macrovasculature, the volume and surface area of each segmented macrovasculature were quantified. Total volume of the CT segmentation of the macrovasculature decreased in MNR + IGF1 (Indirect Exposure) and MNR + IGF1 (Direct Injection) from MNR (p < 0.001, p < 0.05), but no group was different from Control (Fig. 4A). Surface area of segmented macrovasculature increased in MNR from Control (p < 0.05) (Fig. 4B). Surface area decreased in MNR + IGF1 (Indirect Exposure) and MNR + IGF1 (Direct Injection) placentas compared to MNR (p < 0.01, p < 0.05), with no differences from Control.

**Fig. 5.**
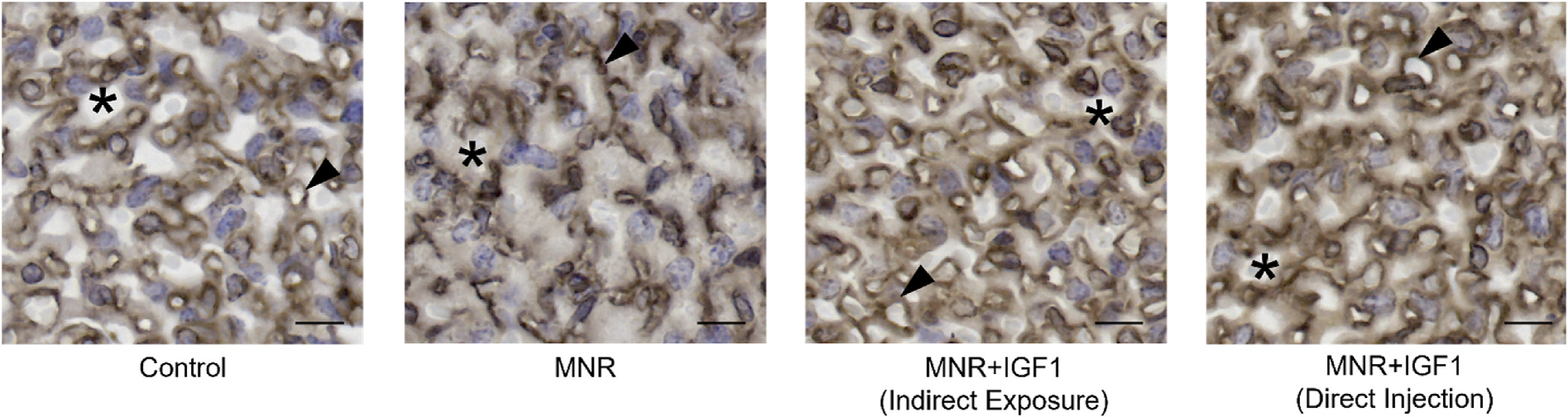
Effects of maternal nutrient restriction (MNR) and repeated *hIGF1* nanoparticle gene therapy (MNR + *hIGF1*) at end of term on placental exchange area. Representative images of immunohistochemistry for cytokeratin and vimentin showing altered placenta exchange area. MNR placentas show a lack of defined structure without clear placental capillary lumens or open maternal blood spaces compared to Control, MNR + IGF1 (Indirect Exposure), and MNR + IGF1 (Direct Injection). Brown: cytokeratin, trophoblast. Black: vimentin, endothelial cells. Stars represent maternal blood spaces; arrow heads represent placental capillaries. Scale bar = 10 μm. Control: Dams: n = 2, placentas: n = 7; MNR: Dams: n = 3, placentas: n = 7; MNR + IGF1: Dams: n = 2, placentas n = 8.

As maternal and fetal circulation could not be separately defined in macrovasculature CT segmentation, each circulation was identified using IHC to analyze the microvasculature of the labyrinthine exchange area. Immunohistochemistry against Cytokeratin allowed identification of labyrinthine trophoblast and vimentin to identify stroma and placental capillaries. The structure of the labyrinth showed a lack of defined maternal blood spaces in MNR compared to Control (Fig. 5). Placental capillaries were also collapsed with indistinct lumens. This implied an increase in interhaemal distance compared to Control whereas MNR + IGF1 (Indirect Exposure) and MNR + IGF1 (Direct Injection) both appeared similar to Control with clear lumens and open maternal blood space.

**Fig. 6.**
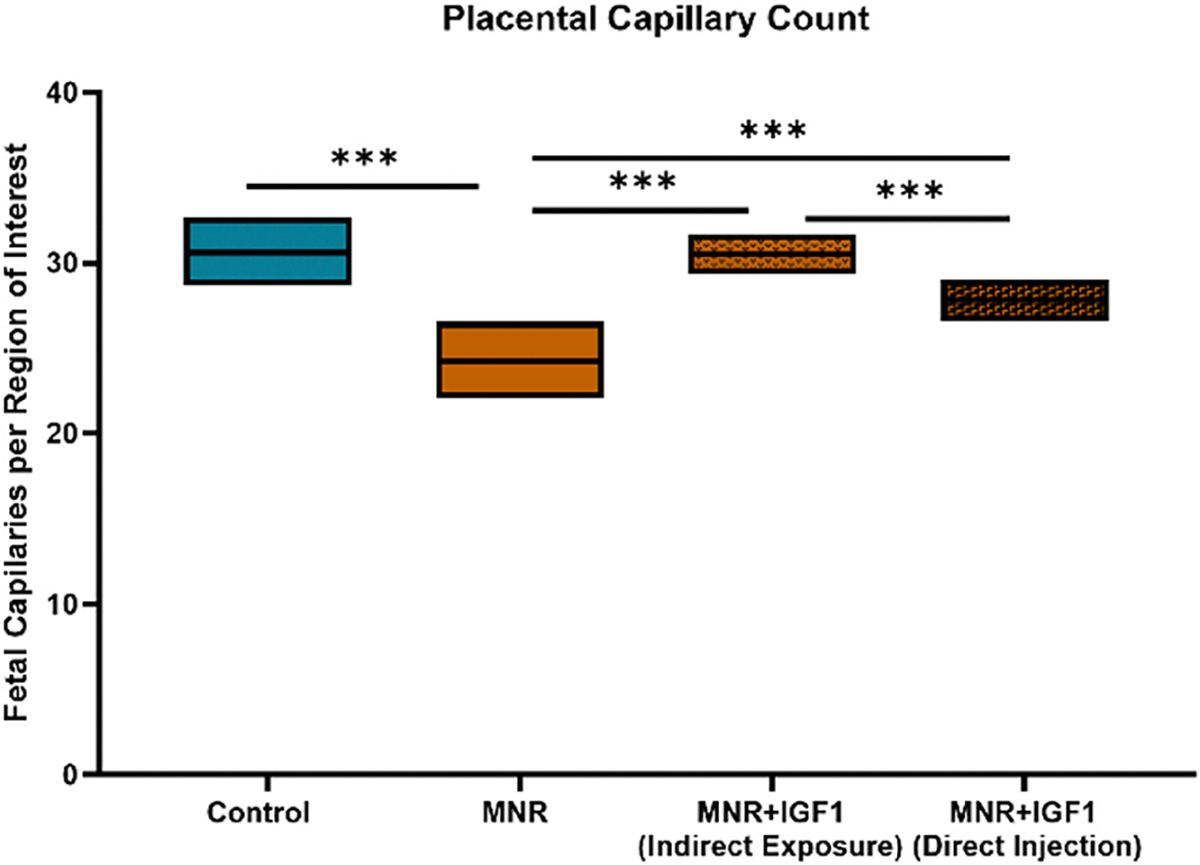
Effects of maternal nutrient restriction (MNR) and repeated *hIGF1* nanoparticle gene therapy (MNR + *hIGF1*) near term on labyrinth capillary count. Placental capillary number decreased in MNR compared to Control MNR + IGF1 (Indirect Exposure), and MNR + IGF1 (Direct Injection). Control: Dams: n = 2, placentas: n = 7; MNR: Dams: n = 3, placentas: n = 7; MNR + IGF1: Dams: n = 2, placentas n = 8. Data are estimated marginal means ± 95 % confidence interval. *P ≤ 0.05; **P ≤ 0.01. ***P ≤ 0.001.

**Fig. 7.**
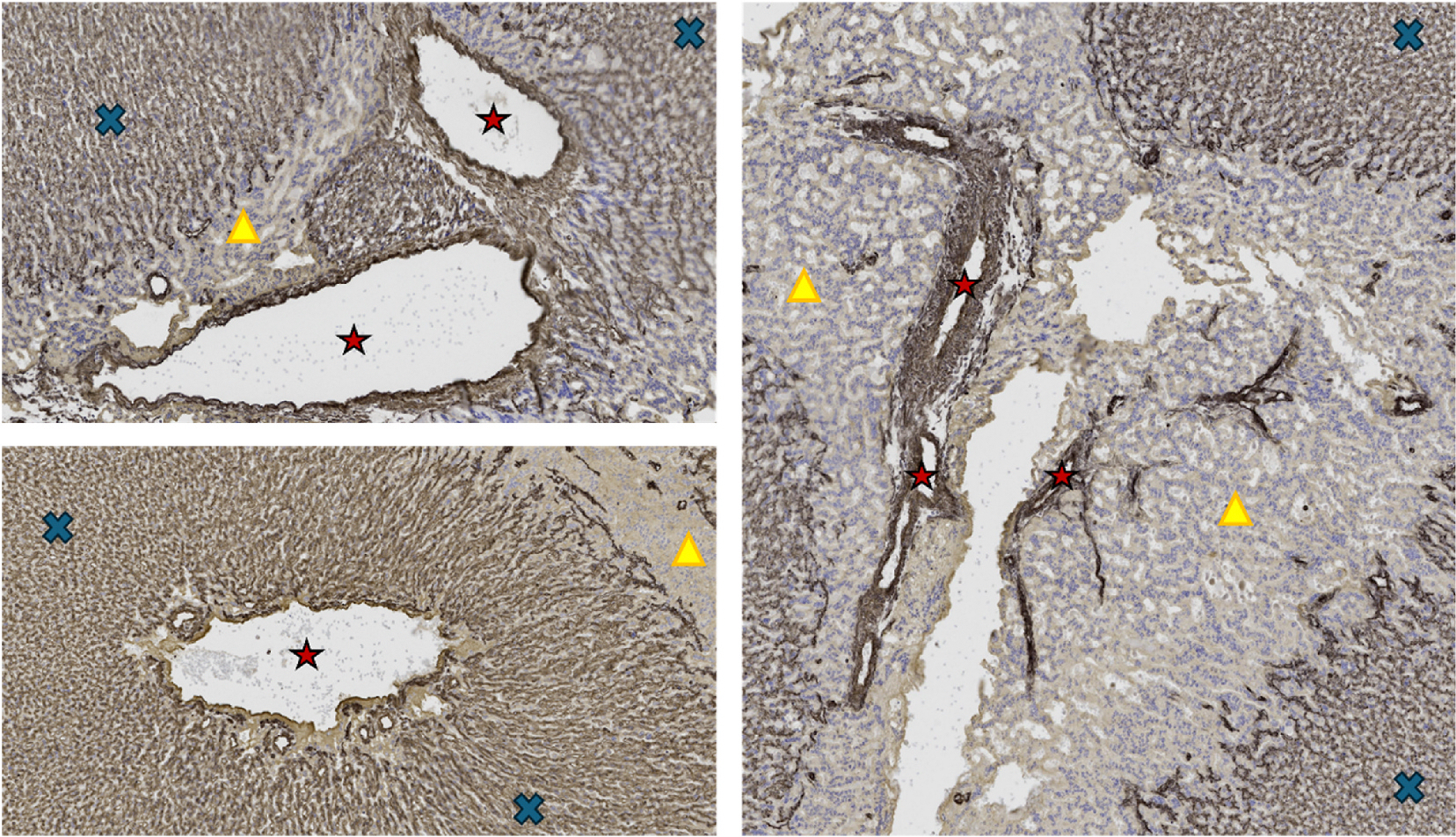
Macrovasculature vessels and microvasculature exchange regions of the placenta. Representative images of immunohistochemistry for cytokeratin and vimentin of Control Placentas. Red stars represent macrovasculature vessels, blue Xs represent microvasculature exchange areas of the labyrinth, yellow triangles represent interlobulum.

Placental capillary count was decreased in MNR compared to Control (Fig. 6). MNR + IGF1 (Indirect Exposure) and MNR + IGF1 (Direct Injection) placental capillary count were both increased from MNR, back to Control levels. For greater clarity and identification of large macrovasculature vessels and microvasculature exchange area vessels within the same image/sample, these areas are identified in a representative Control IHC image (Fig. 7). Images of the placental disc identify macrovasculature vessels, microvasculature exchange areas, and interobulum areas.

## 4. Discussion

There are currently no treatments for fetal growth restriction or placental insufficiency. We have previously shown that nanoparticle-mediated *hIGF1* gene therapy corrects fetal growth, placental weight/efficiency, fetal blood glucose, and maternal and fetal cortisol [23]. In the present study, the aim was to determine the impact of this *hIGF1* treatment on placenta structure to elucidate changes that occur in the placenta labyrinth, leading to better fetal outcomes. Overall, this data demonstrates that the aberrant placental structure occurring with MNR is improved with nanoparticle-mediated *hIGF1* treatment across the latter half of pregnancy.

Doppler ultrasound is frequently used in diagnosis of FGR in humans to assess the blood flow and resistance in the placenta [37–39]. If resistance is high in the uterine arteries or blood flow is low in the umbilical artery, the fetus is at risk for FGR [37,39,40]. P(z) value is a theoretical representation of back pressure in the uteroplacental circulation, and the pulsatility index (PI) assesses the circulatory status or resistance to flow in the vasculature [40]. From these values both maternal and fetal circulation are disrupted in cases of FGR. While unable to get a clear idea of vasculature remodeling across human pregnancy for ethical reasons, animal models offer an opportunity for clearer insights into the structural causes leading to changes in P(z) and PI with placental insufficiency [41].

Using DiceCT, 3D models of the placenta macrovasculature were segmented in a guinea pig model. Quantification of this segmentation revealed increased surface area of the macrovasculature in MNR placentas. From human doppler measures of both umbilical and uterine arteries, it is known that back pressure is a common problem in FGR placentas [2,38,40,42,43]. Increase in surface area of a vessel is generally a response in an effort to lower blood pressure [44]. Forced vasodilation, leading to increased surface area of the vessel, results in decreased blood pressure by distributing the force of the blood over a larger area, reducing the back pressure against the vessel walls, which likely explains the increase in surface area in MNR placentas [43–45]. Volume of the macrovasculature, though not significant, showed the same trend to surface area. As blood volume within a vessel increases, like that in cases of back pressure, the pressure exerted on the vessel walls rises, once again potentially explaining this increase in volume [44]. Repeated nanoparticle-mediated *hIGF1* treatments, however, resulted in surface area and volume no different from Control. The increase in vasculature size in MNR may be due to the increased back pressure of the placental circulation as seen in human cases when the P(z) value increases in FGR, while placentas receiving repeated *hIGF1* treatment did not have aberrant back pressure and therefore no vasodilation of the macrovasculature.

While the maternal from fetal circulation cannot be differentiated in the macrovasculature CT segmentation, investigating the microvasculature (~5–10 μm vessels) of the labyrinth exchange area identified reduced numbers of placental capillaries in MNR significantly decreased from Control placentas. Optimal oxygen diffusion from the maternal to fetal circulation depends on the thickness of tissue, therefore the smaller the interhaemal distance, the easier for oxygen to diffuse [46]. The lack of defined large maternal blood spaces and collapsed placental capillaries separated by larger trophoblast/stroma area in the MNR IHC allude to an increased interhaemal distance compared to Control and MNR + IGF1. At mid-pregnancy there was no change in this interhaemal distance with MNR yet, but *hIGF1* treatment reduced this distance [30].

As gestation progresses, branching of capillaries is essential for placental development to deliver both the increasing amount of nutrients necessary for the exponential growth of the fetus and ensure optimal oxygen transfer to fetal hemoglobin [45,47,48]. Decreased number of placental vessel lumens in MNR placentas suggests decreased angiogenesis from Control. With MNR, as previously demonstrated by Roberts et al. and recapitulated in the current study, placental architecture shows an overall deficit in the exchange area of the placenta elucidating one of the potential main causes of decreased fetal growth/placental insufficiency [22]. With repeated *hIGF1* treatment, however, labyrinthine structure including maternal blood spaces and placental capillaries were restored to Control levels indicating improved angiogenesis and exchange potential.

While previous studies of the guinea pig placenta structure published changes with MNR, this study is the first to employ the DiceCT method to reconstruct the guinea pig placenta macrovasculature [22,49–52]. As previously mentioned, this method is frequently used on museum specimens and/or whole animal tissues to visualize soft tissue and anatomic organization of organ systems [32,34]. Interestingly, in contrast to other measurements previously published, the structural differences and remediation reported here did not vary based on fetal sex. In future studies, the goal is to include doppler measures for uterine and umbilical artery flow to elucidate the similarities in blood flow within the guinea pig placenta to human cases of FGR and assess the effects of *hIGF1* treatment.

## 5. Conclusion

In the present study, changes in the placental macrovasculature and microvasculature exchange area of the labyrinth in MNR placentas recapitulate human placental insufficiency and show improvement following treatment with novel nanoparticle-mediated *hIGF1* gene therapy. These structural improvements likely contribute to the improvement in fetal growth previously published with this therapy.

### 6. Limitations

Limitations of this novel approach include staining times and concentrations dependent on type of tissue (easily corrected with water baths or higher concentration of Lugol’s iodine) and machinery/software improvements. While able to successfully segment the macrovasculature of these placentas, a higher resolution scan on the current GE CT, or an alternative CT such as the Ziess Versa 620 XRM, gives resolution down to 50 nm and a more precise segmentation that includes the microvasculature exchange area and the macrovasculature may be possible in the future. This tool is likely to improve with each software update and version release and is recommended for complex structures such as the placenta vasculature.

## Supplementary Material

Supplementary Material

## Appendix A. Supplementary data

Supplementary data to this article can be found online at https://doi.org/10.1016/j.tria.2025.100424.

## List of abbreviations:

FGR: fetal growth restriction
EFW: estimated fetal weight
DOHaD: developmental origins of health and disease
MNR: maternal nutrient restriction
IGF1: insulin-like growth factor 1
DiceCT: diffusible iodine contrast enhanced computed tomography
IHC: immunohistochemistry
GD: gestational day

## References

[1] Keenan L, 1 in 10 Babies Worldwide are Born Early, with Major Impacts on Health and Survival, Departmental News, World Health Organization, 2023.

[2] Nardozza LM, Caetano AC, Zamarian AC, Mazzola JB, Silva CP, Marçal VM, Lobo TF, Peixoto AB, Araujo Júnior E, Fetal growth restriction: current knowledge, Arch. Gynecol. Obstet. 295 (5) (2017) 1061–1077. 28285426 10.1007/s00404-017-4341-9

[3] de Onis M, Blössner M, Villar J, Levels and patterns of intrauterine growth retardation in developing countries, Eur. J. Clin. Nutr. 52 (Suppl 1) (1998) S5–S15. 9511014

[4] Malacova E, Regan A, Nassar N, Raynes-Greenow C, Leonard H, Srinivasjois R, A WS, Lavin T, Pereira G, Risk of stillbirth, preterm delivery, and fetal growth restriction following exposure in a previous birth: systematic review and meta-analysis, Bjog 125 (2) (2018) 183–192. 28856792 10.1111/1471-0528.14906

[5] Armengaud JB, Yzydorczyk C, Siddeek B, Peyter AC, Simeoni U, Intrauterine growth restriction: clinical consequences on health and disease at adulthood, Reprod. Toxicol. 99 (2021) 168–176. 33049332 10.1016/j.reprotox.2020.10.005

[6] Hanson M, Gluckman P, Developmental origins of noncommunicable disease: population and public health implications, Am. J. Clin. Nutr. 94 (suppl_6) (2011) 1754S–1758S. 21525196 10.3945/ajcn.110.001206

[7] Gagnon R, Placental insufficiency and its consequences, Eur. J. Obstet. Gynecol. Reprod. Biol. 110 (Suppl 1) (2003) S99–S107. 12965097 10.1016/s0301-2115(03)00179-9

[8] Burton GJ, Jauniaux E, Pathophysiology of placental-derived fetal growth restriction, Am. J. Obstet. Gynecol. 218 (2s) (2018) S745–s761. 29422210 10.1016/j.ajog.2017.11.577

[9] Gumina DL, Su EJ, Mechanistic insights into the development of severe fetal growth restriction, Clin. Sci. (Lond.) 137 (8) (2023) 679–695. 37186255 10.1042/CS20220284 PMC10241202

[10] Su EJ, Role of the fetoplacental endothelium in fetal growth restriction with abnormal umbilical artery doppler velocimetry, Am. J. Obstet. Gynecol. 213 (4 Suppl) (2015) S123–S130. 26428491 10.1016/j.ajog.2015.06.038 PMC4592515

[11] Whitley GS, Cartwright JE, Cellular and molecular regulation of spiral artery remodelling: lessons from the cardiovascular field, Placenta 31 (6) (2010) 465–474. 20359743 10.1016/j.placenta.2010.03.002 PMC2882556

[12] Thompson LP, Pence L, Pinkas G, Song H, Telugu BP, Placental hypoxia during early pregnancy causes maternal hypertension and placental insufficiency in the hypoxic Guinea pig Model1, Biol. Reprod. 95 (6) (2016). 10.1095/biolreprod.116.142273 PMC5315426 27806942

[13] Spradley FT, Ge Y, Granger JP, Chade AR, Utero-placental vascular remodeling during late gestation in sprague-dawley rats, Pregnancy Hypertens 20 (2020) 36–43. 32172168 10.1016/j.preghy.2020.02.007 PMC7246036

[14] James JL, Tongpob Y, Srinivasan V, Crew RC, Bappoo N, Doyle B, Gerneke D, Clark AR, Wyrwoll CS, Three-dimensional visualisation of the feto-placental vasculature in humans and rodents, Placenta 114 (2021) 8–13. 34418753 10.1016/j.placenta.2021.08.049

[15] Junaid TO, Brownbill P, Chalmers N, Johnstone ED, Aplin JD, Fetoplacental vascular alterations associated with fetal growth restriction, Placenta 35 (10) (2014) 808–815. 25145956 10.1016/j.placenta.2014.07.013

[16] Junaid TO, Bradley RS, Lewis RM, Aplin JD, Johnstone ED, Whole organ vascular casting and microCT examination of the human placental vascular tree reveals novel alterations associated with pregnancy disease, Sci. Rep. 7 (1) (2017) 4144. 28646147 10.1038/s41598-017-04379-0 PMC5482861

[17] Mess A, The Guinea pig placenta: model of placental growth dynamics, Placenta 28 (8–9) (2007) 812–815. 17382996 10.1016/j.placenta.2007.02.005

[18] Kaufmann P, Benirschke K, Comparative placentation: guinea pig. http://placentation.ucsd.edu/guinea.htm, 2004.

[19] Morrison JL, Botting KJ, Darby JRT, David AL, Dyson RM, Gatford KL, Gray C, Herrera EA, Hirst JJ, Kim B, Kind KL, Krause BJ, Matthews SG, Palliser HK, Regnault TRH, Richardson BS, Sasaki A, Thompson LP, Berry MJ, Guinea pig models for translation of the developmental origins of health and disease hypothesis into the clinic, J. Physiol. 596 (23) (2018) 5535–5569. 29633280 10.1113/JP274948 PMC6265540

[20] Elias AA, Ghaly A, Matushewski B, Regnault TRH, Richardson BS, Maternal nutrient restriction in Guinea pigs as an animal model for inducing fetal growth restriction, Reprod. Sci. 23 (2) (2016) 219–227. 26342049 10.1177/1933719115602773

[21] Elias AA, Maki Y, Matushewski B, Nygard K, Regnault TRH, Richardson BS, Maternal nutrient restriction in guinea pigs leads to fetal growth restriction with evidence for chronic hypoxia, Pediatr. Res. 82 (1) (2017) 141–147. 28376077 10.1038/pr.2017.92

[22] Roberts CT, Sohlstrom A, Kind KL, Earl RA, Khong TY, Robinson JS, Owens PC, Owens JA, Maternal food restriction reduces the exchange surface area and increases the barrier thickness of the placenta in the guinea-pig, Placenta 22 (2–3) (2001) 177–185. 11170822 10.1053/plac.2000.0602

[23] Davenport BN, Wilson RL, Williams AA, Jones HN, Placental nanoparticle-mediated IGF1 gene therapy corrects fetal growth restriction in a Guinea pig model, Gene Ther. 32 (3) (2025) 255–265. 39627510 10.1038/s41434-024-00508-3 PMC12105984

[24] Elias AA, Ghaly A, Matushewski B, Regnault TR, Richardson BS, Maternal nutrient restriction in Guinea pigs as an animal model for inducing fetal growth restriction, Reprod. Sci. 23 (2) (2016) 219–227. 26342049 10.1177/1933719115602773

[25] Randhawa RS, The insulin-like growth factor system and fetal growth restrictionn, Pediatr. Endocrinol. Rev. 6 (2) (2008) 235–240. 19202510

[26] Martín-Estal I, de la Garza RG, Castilla-Cortázar I, Intrauterine growth retardation (IUGR) as a novel condition of insulin-like growth Factor-1 (IGF-1) deficiency, Rev. Physiol. Biochem. Pharmacol. 170 (2016) 1–35. 26634242 10.1007/112_2015_5001

[27] Bulmer JN, Burton GJ, Collins S, Cotechini T, Crocker IP, Croy BA, Cvitic S, Desforges M, Deshpande R, Gasperowicz M, Groten T, Haugen G, Hiden U, Host AJ, Jirkovska M, Kiserud T, Konig J, Leach L, Murthi P, Pijnenborg R, Sadekova ON, Salafia CM, Schlabritz-Loutsevitch N, Stanek J, Wallace AE, Westermeier F, Zhang J, Lash GE, IFPA meeting 2011 workshop report II: angiogenic signaling and regulation of fetal endothelial function; placental and fetal circulation and growth; spiral artery remodeling, Placenta 33 (Suppl) (2012) S9–S14. 22177322 10.1016/j.placenta.2011.11.014

[28] Hiden U, Glitzner E, Hartmann M, Desoye G, Insulin and the IGF system in the human placenta of normal and diabetic pregnancies, J.Anat. 215 (1) (2009) 60–68. 19467150 10.1111/j.1469-7580.2008.01035.x PMC2714639

[29] Davenport BN, Jones HN, Wilson RL, Placental treatment with insulin-like growth factor 1 via nanoparticle differentially impacts vascular remodeling factors in guinea pig sub-placenta/decidua, Front. Physiol. 13 (2023). 10.3389/fphys.2022.1055234 PMC9845775 36685211

[30] Wilson RL, Lampe K, Gupta MK, Duvall CL, Jones HN, Nanoparticle-mediated transgene expression of insulin-like growth factor 1 in the growth restricted guinea pig placenta increases placenta nutrient transporter expression and fetal glucose concentrations, Mol. Reprod. Dev. 89 (11) (2022) 540–553. 36094907 10.1002/mrd.23644 PMC10947605

[31] Wilson RL, Stephens KK, Lampe K, Gupta MK, Duvall CL, Jones HN, Nanoparticle-mediated transgene expression of insulin-like growth factor 1 in the guinea pig placenta differentially affects fetal liver gene expression depending on maternal nutrient status, bioRxiv (2021), 2021.06.24.449769.

[32] Gray JA, Gignac PM, Stanley EL, The first full body diffusible iodine-based contrast-enhanced computed tomography dataset and teaching materials for a member of the Testudines, Anat. Rec. 307 (3) (2024) 535–548. 10.1002/ar.25282 37409685

[33] Callahan S, Crowe-Riddell JM, Nagesan RS, Gray JA, Davis Rabosky AR, A guide for optimal iodine staining and high-throughput diceCT scanning in snakes, Ecol. Evol. 11 (17) (2021) 11587–11603. 34522326 10.1002/ece3.7467 PMC8427571

[34] Gignac PM, Kley NJ, Clarke JA, Colbert MW, Morhardt AC, Cerio D, Cost IN, Cox PG, Daza JD, Early CM, Echols MS, Henkelman RM, Herdina AN, Holliday CM, Li Z, Mahlow K, Merchant S, Müller J, Orsbon CP, Paluh DJ, Thies ML, Tsai HP, Witmer LM, Diffusible iodine-based contrast-enhanced computed tomography (diceCT): an emerging tool for rapid, high-resolution, 3-D imaging of metazoan soft tissues, J. Anat. 228 (6) (2016) 889–909. 26970556 10.1111/joa.12449 PMC5341577

[35] Dawood Y, Hagoort J, Siadari BA, Ruijter JM, Gunst QD, Lobe NHJ, Strijkers GJ, de Bakker BS, van den Hoff MJB, Reducing soft-tissue shrinkage artefacts caused by staining with Lugol’s solution, Sci. Rep. 11 (1) (2021) 19781. 34611247 10.1038/s41598-021-99202-2 PMC8492742

[36] Schindelin J, Arganda-Carreras I, Frise E, Kaynig V, Longair M, Pietzsch T, Preibisch S, Rueden C, Saalfeld S, Schmid B, Tinevez J-Y, White DJ, Hartenstein V, Eliceiri K, Tomancak P, Cardona A, Fiji: an open-source platform for biological-image analysis, Nat. Methods 9 (7) (2012) 676–682. 22743772 10.1038/nmeth.2019 PMC3855844

[37] Audette MC, Kingdom JC, Screening for fetal growth restriction and placental insufficiency, Semin. Fetal Neonatal Med. 23 (2) (2018) 119–125. 29221766 10.1016/j.siny.2017.11.004

[38] Bamfo JE, Odibo AO, Diagnosis and management of fetal growth restriction, J Pregnancy 2011 (2011) 640715. 21547092 10.1155/2011/640715 PMC3087156

[39] Rizzo G, Mappa I, Bitsadze V, Słodki M, Khizroeva J, Makatsariya A, D’Antonio F, Role of doppler ultrasound at time of diagnosis of late-onset fetal growth restriction in predicting adverse perinatal outcome: prospective cohort study, Ultrasound Obstet. Gynecol. 55 (6) (2020) 793–798. 31343783 10.1002/uog.20406

[40] Maseliene T, Zukiene G, Laurinaviciene A, Breskuviene D, Ramasauskaite D, Dzenkeviciute V, Alterations in maternal cardiovascular parameters and their impact on uterine and fetal circulation in hypertensive pregnancies and fetal growth restriction, International Journal of Cardiology Cardiovascular Risk and Prevention 22 (2024) 200316. 39206138 10.1016/j.ijcrp.2024.200316 PMC11350454

[41] Wardinger JE, Ambati S, Placental Insufficiency, StatPearls, StatPearls Publishing Copyright © 2022, StatPearls Publishing LLC., Treasure Island (FL), 2022.

[42] Figueras F, Caradeux J, Crispi F, Eixarch E, Peguero A, Gratacos E, Diagnosis and surveillance of late-onset fetal growth restriction, Am. J. Obstet. Gynecol. 218 (2, Supplement) (2018). S790–S802.e1. 29422212 10.1016/j.ajog.2017.12.003

[43] Jones S, Bischof H, Lang I, Desoye G, Greenwood SL, Johnstone ED, Wareing M, Sibley CP, Brownbill P, Dysregulated flow-mediated vasodilatation in the human placenta in fetal growth restriction, J. Physiol. 593 (14) (2015) 3077–3092. 25920377 10.1113/JP270495 PMC4532528

[44] Ramanlal R GV, Physiology, vasodilation, StatPearls (2025).

[45] Zygmunt M, Herr F, Münstedt K, Lang U, Liang OD, Angiogenesis and vasculogenesis in pregnancy, Eur. J. Obstet. Gynecol. Reprod. Biol. 110 (Suppl 1) (2003) S10–S18. 12965086 10.1016/s0301-2115(03)00168-4

[46] Ansari T, Fenlon S, Pasha S, O’Neill B, Gillan JE, Green CJ, Sibbons PD, Morphometric assessment of the oxygen diffusion conductance in placentae from pregnancies complicated by intra-uterine growth restriction, Placenta 24 (6) (2003) 618–626. 12828920 10.1016/s0143-4004(03)00044-4

[47] Arroyo JA, Winn VD, Vasculogenesis and angiogenesis in the IUGR placenta, Semin. Perinatol. 32 (3) (2008) 172–177. 18482617 10.1053/j.semperi.2008.02.006

[48] Chen DB, Zheng J, Regulation of placental angiogenesis, Microcirculation 21 (1) (2014) 15–25. 23981199 10.1111/micc.12093 PMC5589442

[49] Dockery P, Bermingham J, Jenkins D, Structure-function relations in the human placenta, Biochem. Soc. Trans. 28 (2) (2000) 202–208. 10816128 10.1042/bst0280202

[50] Enders AC, Blankenship TN, Comparative placental structure, Adv. Drug Deliv. Rev. 38 (1) (1999) 3–15. 10837743 10.1016/s0169-409x(99)00003-4

[51] Guttmacher AE, Maddox YT, Spong CY, The human placenta project: placental structure, development, and function in real time, Placenta 35 (5) (2014) 303–304. 24661567 10.1016/j.placenta.2014.02.012 PMC3999347

[52] Sferruzzi-Perri AN, Owens JA, Standen P, Roberts CT, Maternal insulin-like growth Factor-II promotes placental functional development via the type 2 IGF receptor in the Guinea pig, Placenta 29 (4) (2008) 347–355. 18339421 10.1016/j.placenta.2008.01.009

