# Supplementary material for: Aberrant placental structure is corrected with repeated nanoparticle-mediated IGF1 treatments in a Guinea pig model of fetal growth restriction
Source: Transl Res Anat. Author manuscript; Available in PMC 2026 Mar 21. (PMC13003953; doi:10.1016/j.tria.2025.100424)
Supplement: Supplementary Material [file NIHMS2152921-supplement-Supplementary_Material.docx]

**Submission Checklist**

- One author has been designated as the corresponding author and their full contact details (email address, full postal address and phone numbers) have been provided.

**b.davenport@ufl.edu**

**1354 Center Dr. M552 PO Box 100274, Gainesville, FL 32610**

**352-392-3719**

- All files have been uploaded, including keywords, figure captions and tables (including a title, description and footnotes) included.

**All files have been uploaded**

- Spelling and grammar checks have been carried out.

**All spelling and grammar has been checked**

- All references in the article text are cited in the reference list and vice versa.

**All articles have been cited**

- Permission has been obtained for the use of any copyrighted material from other sources, including the Web.

**All necessary permissions have been obtained**

- For gold open access articles, all authors understand that they are responsible for payment of the article publishing charge (APC) if the manuscript is accepted. Payment of the APC may be covered by the corresponding author's institution, or the research funder.
